# A comparison of cardiovascular magnetic resonance and single photon emission computed tomography (SPECT) perfusion imaging in left main stem or equivalent coronary artery disease: a CE-MARC substudy

**DOI:** 10.1186/s12968-017-0398-7

**Published:** 2017-11-06

**Authors:** James R. J. Foley, Ananth Kidambi, John D. Biglands, Neil Maredia, Catherine J. Dickinson, Sven Plein, John P. Greenwood

**Affiliations:** 1 Multidisciplinary Cardiovascular Research Centre & The Division of Biomedical Imaging, Leeds Institute of Cardiovascular and Metabolic Medicine, University of Leeds, Leeds, LS2 9JT UK; 2 Cardiology Department, Leeds General Infirmary, Leeds, UK

**Keywords:** Coronary artery disease, Left main stem disease, Cardiovascular magnetic resonance, SPECT, Quantitative perfusion

## Abstract

**Background:**

Assessment of left main stem (LMS) stenosis has prognostic and therapeutic implications. Data on assessment of LMS disease by cardiovascular magnetic resonance (CMR) and single photon emission computed tomography (SPECT) are limited. CE-MARC is the largest prospective comparison of CMR and SPECT against quantitative invasive coronary angiography (QCA) for detection of coronary artery disease (CAD), and provided the framework for this evaluation. The aims of this study were to compare diagnostic accuracy of visual and quantitative perfusion CMR to SPECT in patients with LMS stable CAD.

**Methods:**

Fifty-four patients from the CE-MARC study were included: 27 (4%) with significant LMS or LMS-equivalent disease on QCA, and 27 age/sex-matched patients with no flow-limiting CAD. All patients underwent multi-parametric CMR, SPECT and QCA. Performance of visual and quantitative perfusion CMR by Fermi-constrained deconvolution to detect LMS disease was compared with SPECT.

**Results:**

Of 27 patients in the LMS group, 22 (81%) had abnormal CMR and 16 (59%) had abnormal SPECT. All patients with abnormal CMR had abnormal perfusion by visual analysis. CMR demonstrated significantly higher area under the curve (AUC) for detection of disease (0.95; 0.85–0.99) over SPECT (0.63; 0.49–0.76) (*p* = 0.0001). Global mean stress myocardial blood flow (MBF) by CMR in LMS patients was significantly lower than controls (1.77 ± 0.72 ml/g/min vs. 3.28 ± 1.20 ml/g/min, *p* < 0.001). MBF of <2.08 ml/g/min had sensitivity of 78% and specificity of 85% for diagnosis of LMS disease, with an AUC (0.87; 0.75–0.94) not significantly different to visual CMR analysis (*p* = 0.18), and more accurate than SPECT (*p* = 0.003).

**Conclusion:**

Visual stress perfusion CMR had higher diagnostic accuracy than SPECT to detect LMS disease. Quantitative perfusion CMR had similar performance to visual CMR perfusion analysis.

## Background

Left main stem (LMS) coronary artery disease (CAD) is found in approximately 5% of patients with stable angina and in approximately 7% of patients presenting with an acute myocardial infarction [1]. Significant LMS disease is typically defined as a stenosis of ≥50% and LMS equivalent as ≥70% stenosis of both the proximal left anterior descending artery (LAD) and proximal circumflex artery (LCx). Significant LMS disease is associated with poor clinical outcomes, with an untreated 3-year survival of 50% in those with >50% stenosis dropping to 41% in those with stenosis >70% [2, 3]. Several studies have demonstrated survival benefit for revascularisation of significant LMS stenosis [4, 5]. Thus, accurate detection and functional assessment of the degree of LMS stenosis has both important prognostic and therapeutic implications.

Patients evaluated for suspected CAD frequently undergo functional imaging, which may include single-photon emission computed tomography (SPECT) or cardiovascular magnetic resonance (CMR) imaging. A normal myocardial perfusion study by either of these techniques is associated with an excellent long-term prognosis [6–8]. Published data on the utility of SPECT for the diagnosis of LMS disease are limited, with variable diagnostic accuracy reported [9–12]. Equally, the diagnostic accuracy of stress perfusion CMR is poorly established in LMS disease.

The Clinical Evaluation of MAgnetic Resonance imaging in Coronary heart disease (CE-MARC) study [13, 14] was a large prospective study of patients with suspected CAD; 752 patients were enrolled and all were scheduled to undergo CMR, SPECT and the reference standard invasive coronary angiography. Using the CE-MARC dataset, we hypothesised that CMR would have a greater diagnostic accuracy than SPECT for the detection of LMS or LMS equivalent CAD, and that quantitative CMR perfusion analysis would improve diagnostic discrimination compared to visual analysis.

## Methods

### Subjects

All patients with LMS disease ≥50%, and left main equivalent (≥70% stenosis of proximal LAD and LCx arteries) by quantitative coronary angiography (QCA) were selected from the CE-MARC population, together with an equal number of control patients without significant stenosis on X-ray angiography. The control patients were independently matched to the LMS group for age, sex and cardiovascular risk factors. The inclusion criteria and full imaging protocol for CE-MARC have been previously reported [14]. In brief, inclusion criteria were: stable chest pain thought to be angina pectoris, at least one cardiovascular disease risk factor, suitability for coronary revascularisation if required and in sinus rhythm. Exclusion criteria were: previous coronary artery bypass surgery, evidence of crescendo angina or acute coronary syndrome, contraindication to CMR imaging or adenosine infusion, and chronic renal failure. The study was performed in accordance with the Declaration of Helsinki (October 2000), with all patients providing informed written consent. The study protocol and other relevant documentation had been approved by the National Research Ethics Committee.

### CMR protocol

Patients underwent perfusion-CMR on a 1.5 T scanner (Philips Medical Systems, Best, The Netherlands) equipped with “Master” gradients (30 mT/m peak gradient, 150 mT/m/ms slew rate) and a five-element cardiac phased-array receiver coil. Stress perfusion imaging was performed using intravenous adenosine (140mcg/kg/min) infused for 4 min. Perfusion imaging was performed every heartbeat during the first-pass in 3 short-axis imaging planes, representing the basal, midventricular, and apical myocardial segments. Images were acquired by using a T1-weighted saturation recovery turbo field-echo imaging sequence*,* using a shared (non–slice-selective) saturation pulse. A bolus of 0.05 mmol/kg gadopentetate dimeglumine [Gd-DTPA], (Magnevist, Bayer Schering Health Care Limited, UK) followed by a 15 ml saline flush was administered at 5 ml/s into an antecubital vein by a power injector (Medrad Spectris Solaris, Medrad Inc., Warrendale, Pennsylvania, USA). Resting myocardial perfusion was then assessed and the data obtained with identical parameters as for the resting perfusion acquisition. The CMR protocol also included cine imaging for assessment of left ventricular (LV) function and late gadolinium enhancement imaging (LGE) [14].

### SPECT protocol

SPECT radionuclide imaging was carried out on a dedicated cardiac gamma camera (MEDISO Cardio-C, Budapest, Hungary), using a two-day scanning protocol, the radioisotope tracer ^99m^Tc tetrofosmin (Myoview), with a standard dose of 400 MBq, weight-adjusted to a maximum of 600 MBq, per examination. Stress and rest ECG-gated SPECT images were acquired. The stress imaging protocol was performed using intravenous adenosine (140mcg/kg/min) for 4 min followed by isotope injection to minimise variation between SPECT and CMR [14].

### X-ray angiography

All patients underwent invasive X-ray coronary angiography by a cardiologist (blinded to SPECT and CMR results).

### CMR analysis

The methods for the visual analysis of CMR in CE-MARC have been described previously [14]. As per the original analysis, CMR was deemed positive if one or more abnormality of perfusion, wall motion abnormality or scar was present [13, 14].

**Fig. 1:**
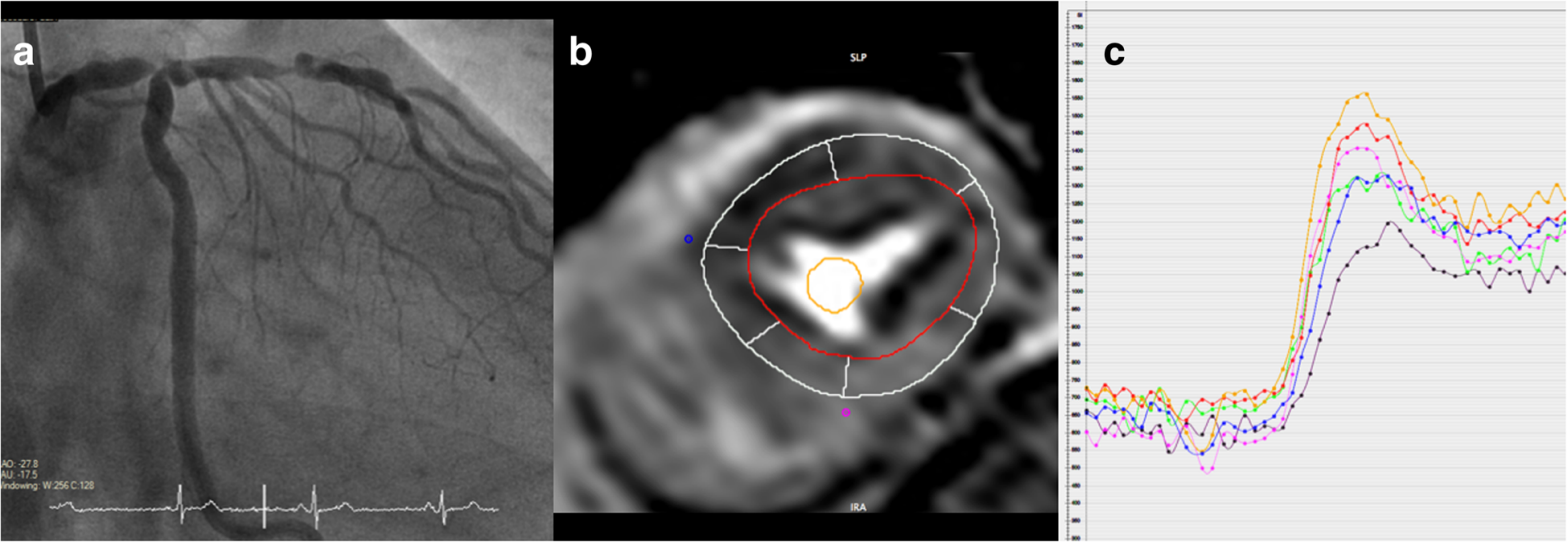
Image panel showing angiography and CMR perfusion of patient with left main stem (LMS) disease. Panel **a** shows angiography with a critical distal LMS lesion. The corresponding mid-slice CMR stress perfusion (**b**) demonstrates a perfusion defect in septum, anterior and lateral wall. Myocardial curves (**c**) of the same mid ventricular slice demonstrates hypoperfusion in the segments subtended by the LMS. Orange and red lines represent the inferior and infero-lateral segments respectively and show higher signal intensity corresponding with no hypoperfusion in these segments

For quantitative perfusion analysis, perfusion CMR data were exported in DICOM format and post-processed off-line using the software cvi42, (version 5.1.0, Circle Cardiovascular Imaging, Calgary, Alberta, Cananda.) Contours depicting the myocardium and a region within the LV blood pool were drawn manually (Fig. 1). These contours were copied to all time frames and manually adjusted for breathing motion by using rigid translation. The myocardium was subdivided into six circumferentially equidistant regions in the basal and middle sections and four in the apical section according to the standard American Heart Association (AHA) model [15].

Quantitative perfusion parameters were calculated using in-house software written in Matlab (Mathworks, Natick, Massachusetts, USA) [16]. Myocardial blood flow (MBF) was estimated using Fermi-constrained deconvolution [17]. Blood pool and myocardial curves were converted to contrast agent concentrations assuming a linear relationship between signal intensity and concentration as previously described [16]. An assumed native blood T_1_ value of 1435 ms and a contrast agent relaxivity of 4.3 s^−1^·mM^−1^ was used. The arterial input function was taken from the basal slice (which had the shortest preparation delay). Concentration curves were baseline subtracted, corrected for temporal shifts between the arterial input function and the myocardial curves and limited to the first pass of contrast through the left ventricle using previously described automated methods [16, 18]. Myocardial perfusion reserve (MPR) was calculated as the ratio of stress MBF to rest MBF. Segmental MBF and MPR were averaged to produce per-patient indices for statistical analysis. This was performed with 16 segments to give a global myocardial value, and separately for segments in the LMS territory. The LMS territory comprised segments 1, 2, 5–8, 11–14 and 16 [15]. A quantitative SSS was produced by applying the optimal MBF value derived by Youden’s index (as detailed in the statistical methods) to the MBF generated in each of the 16 segments for each patient.

### X-ray angiography analysis

X-ray angiography images were analysed by two cardiologists experienced in invasive coronary angiography. QCA analysis was performed off-line using QCAPlus software (Sanders Data Systems, Palo Alto, California, USA). For all LMS patients, visual and quantitative analysis of the invasive angiogram were concordant.

### SPECT analysis

SPECT data sets were analysed in a blinded manner, simultaneously by a cardiologist with >10 years’ experience in nuclear cardiology and an experienced medical physicist. Evidence of ischaemia by visual comparison of rest/stress perfusion scans, based on the standard 17-segment AHA model, was performed. Additionally, evidence of ischaemia by semi-quantitative scoring (using the QPS 20 segment) (QPS, Cedars-Sinai Medical Center, Los Angeles, California, USA**)** was also performed. Non-perfusion markers of significant CAD, such as transient LV dilatation (TID) and increased right ventricular uptake were also taken in to consideration as felt appropriate by the reporting team.

### Data analysis and statistics

Statistical analysis was performed using commercially available software (SPSS, version 22.0, International Business Machines, Armonk, New York, USA). Two-sided *p* values ≤0.05 were considered to be statistically significant. Data were compared using Student’s *t*-test for continuous variables and Fisher’s Exact test for proportions, independent samples *t* tests and Pearson’s correlation coefficients as necessary. Normality for MBF values in the normal comparison group was evaluated using a Q-Q plot and Shapiro-Wilk test. Receiver operating characteristic (ROC) curve analysis for diagnostic tests were compared using the method described by DeLong et al. [19]. For quantitative perfusion analysis, the optimal sensitivity and specificity of quantitative parameters were derived by calculating Youden’s index [20]. The sensitivity and specificity and ROC analysis were based on the 54 patients.

## Results

### Visual analysis

Twenty-seven (4%) patients of the 729 patients that received invasive angiography from CE-MARC were identified to have LMS or LMS equivalent disease by invasive angiography. Twenty-two patients had true LMS disease and 5 patients had LMS equivalent disease. Patient characteristics are shown in Table 1.

**Table 1:** Patient characteristics

Patient characteristic	LMS	Controls	*P*
N	27	27	
Age (years)	65 ± 7	64 ± 6	0.45
Male	23 (85%)	23 (85%)	1.0
Body mass index (kg/m^2^)	27.5 ± 3.89	27.0 ± 2.87	0.60
Current smoker	5 (19%)	4 (15%)	1.0
Blood pressure (mmHg)	134/74 ± 20/10	140/76 ± 19/7	0.27/0.43
Hypertension	12 (44%)	17 (62%)	0.27
Total cholesterol (mmol/L)	5.3 ± 1.4	4.8 ± 1.2	0.25
Diabetes mellitus	5 (19%)	5 (19%)	1.0
Family history of CAD^a^	14 (52%)	13 (48%)	1.0
Significant CAD^a^			
- LMS	22 (81%)	0 (0%)	<0.001
- LAD	17 (63%)	0 (0%)	<0.001
- LCx	11 (41%)	0 (0%)	<0.001
- RCA	11 (41%)	0 (0%)	<0.001

Data as mean ± SD or n (%)

^a^ *CAD* coronary artery disease, *LAD* left anterior descending coronary artery, *LCx* left circumflex coronary artery, *RCA* right coronary artery

**Fig. 2:**
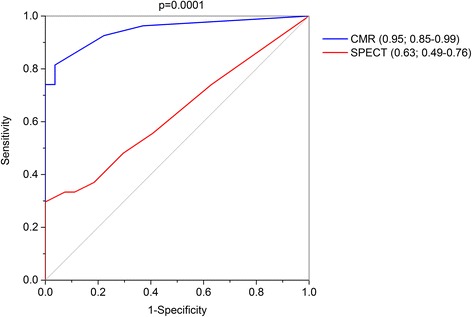
ROC curves for visual summed stress scores for CMR and single photon emission computed tomography (SPECT). Numbers in parentheses indicates area under the curve (AUC) with 95% confidence intervals

All patients had completed CMR, SPECT and angiography studies. Detection rates for CAD by both CMR and SPECT are shown in Table 2. Multi-parametric CMR detected evidence of CAD in a non-significantly higher proportion of patients with LMS disease than SPECT (81% vs. 59%, *p* = 0.14). All patients with abnormal multi-parametric CMR also had abnormal perfusion CMR by visual analysis. One patient was deemed a false negative by SPECT that had 1 segment of inferior ischaemia. For CMR, the average SSS for LMS patients was 13.0 ± 9.5, and for controls 0.67 ± 1.0 (*p* < 0.001). For SPECT, the average SSS for LMS patients was 5.15 ± 6.5, and for controls 1.93 ± 2.3 (*p* = 0.02). ROC analysis demonstrated a significantly higher area under the curve (AUC) for detection of LMS disease by visual CMR analysis compared to SPECT (0.95 vs. 0.63; *p* = 0.0001, Fig. 2).

**Table 2:** Imaging findings

Imaging finding	LMS	Control	*P*
CMR			
- RWMA^a^ positive	17 (63%)	0 (0%)	<0.001
- FPP positive	22 (81%)	1 (4%)	<0.001
- LGE positive	15 (56%)	0 (0%)	<0.001
- Overall positive	22 (81%)	1 (4%)	<0.001
SPECT			
- RWMA positive	10 (37%)	6 (22%)	0.37
- Fixed defect	6 (22%)	5 (19%)	1.0
- Inducible defect	17 (63%)	4 (15%)	<0.001
- TID	1 (4%)	1 (4%)	1.0
- RV uptake	17 (63%)	14 (52%)	0.58
- Overall positive	16 (59%)	3 (11%)	<0.001

Data as n (%)

^a^ *RWMA* regional wall motion abnormality, FPP first pass perfusion, LGE late gadolinium enhancement, TID left ventricular transient ischaemic dilatation, RV right ventricular isotope uptake

### Quantitative CMR perfusion analysis

Table 3 shows the results of the quantitative CMR perfusion analysis. Mean stress MBF and mean MPR were both significantly lower in LMS patients compared to controls (*p* < 0.001); resting MBF was similar between the LMS and control groups (*p* = 0.14).

**Table 3:** Quantitative CMR perfusion analysis

	LMS	Control	*P*-value
Global stress MBF	1.77 ± 0.72	3.28 ± 1.20	<0.001
Global rest MBF	1.28 ± 0.42	1.48 ± 0.55	0.14
Global MPR	1.42 ± 0.44	2.31 ± 0.76	<0.001
LMS territory stress MBF	2.03 ± 0.77	3.38 ± 1.15	<0.001
LMS territory rest MBF	1.42 ± 0.36	1.54 ± 0.56	0.36
LMS territory MPR	1.53 ± 0.44	2.34 ± 0.64	<0.001

MBF values are in ml/g/min

*MBF* myocardial blood flow, *MPR* myocardial perfusion reserve

**Fig. 3:**
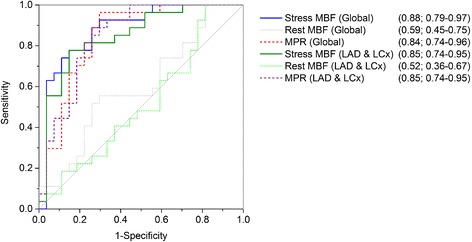
ROC curves for CMR quantitative perfusion results. Numbers in parentheses indicates AUC with 95% confidence intervals

**Fig. 4:**
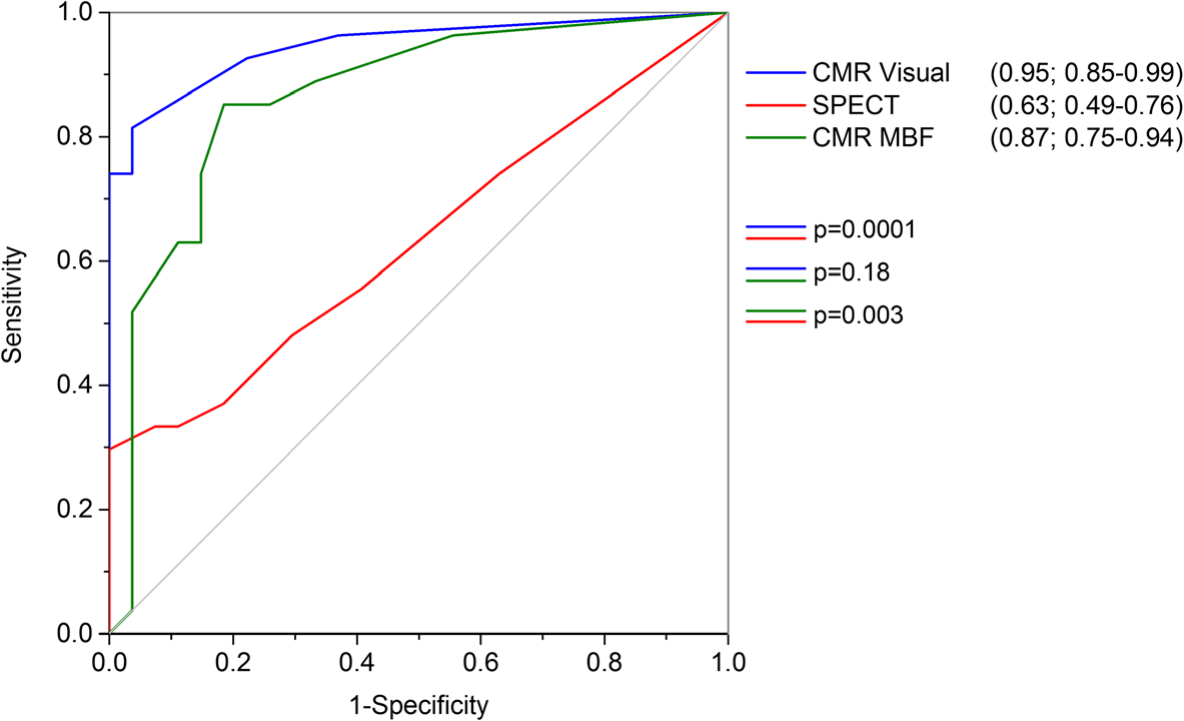
ROC curves for quantitative summed stress score for MBF, visual CMR and SPECT. Numbers in parentheses indicates AUC with 95% confidence intervals

ROC analysis (Fig. 3) demonstrated the highest AUC (0.88) for global MBF as an association with LMS disease. Global MBF of <2.08 ml/g/min was associated with a sensitivity of 78% and specificity of 85% for diagnosis of significant LMS disease. A quantitative SSS was produced using this value; this score had an AUC not significantly different to CMR visual analysis (*p* = 0.18), and more accurate than SPECT (*p* = 0.003, Fig. 4).

Table 4 shows sensitivity, specificity and predictive values for overall visual analysis by multi-parametric CMR and SPECT, and quantitative analysis by CMR global MBF.

**Table 4:** Sensitivity, specificity and predictive values for detection of IHD in LMS patients by visual CMR analysis, SPECT and quantitative CMR

	Sensitivity	Specificity	PPV*	NPV*
CMR Visual	81	96	48	99
CMR MBF	78	85	18	98
SPECT	63	89	19	98

Predictive values are corrected based on the prevalence of LMS disease in the CE-MARC population. *PPV positive predictive value, NPV negative predictive value

## Discussion

This exploratory analysis of the CE-MARC study has demonstrated the diagnostic accuracy of CMR and SPECT in the setting of LMS (or equivalent) CAD. The main finding is that in patients with stable suspected CAD, CMR first-pass perfusion imaging as part of a multi-parametric protocol more accurately detected evidence of CAD in LMS patients than SPECT. Additionally, quantitative CMR perfusion showed high diagnostic accuracy for the detection of LMS disease with global MBF as the most diagnostic, however quantitative perfusion did not outperform visual CMR perfusion analysis.

CMR is established as a cost effective investigation with high diagnostic accuracy compared to SPECT for the diagnosis of suspected CAD [13, 21–23]. Previous data on the diagnostic accuracy of SPECT and CMR in LMS disease are sparse. Thus far there are no studies specifically investigating the diagnostic accuracy of CMR for LMS disease. The MR-IMPACT study [24], a multicentre comparison of CMR and SPECT in 234 patients, included eight patients with LMS disease, while MR-IMPACT II analysed 465 patients of which 14 had LMS disease [22]; in neither of these studies were patients with LMS disease separately analysed. The majority of studies validating CMR perfusion techniques have less than five LMS patients, effectively precluding meaningful analysis of this subset. In contrast, the CE-MARC study had a LMS population of sufficient size to allow reasonable conclusions to be drawn [13]. SPECT studies of LMS disease have largely been un-blinded, retrospective and derived from angiographic databases [9–11]; in this context referral bias potentially leads to an over-estimation of the sensitivity of SPECT for the detection of LMS disease [10], as the false negative SPECT scans go unevaluated.

Non-invasive detection of CAD is clinically useful to both determine the presence of clinically significant disease and to estimate the severity and extent of disease. The classical finding of an inducible perfusion abnormality involving both the LAD and LCx coronary artery territories was not robustly seen in LMS patients by either CMR or SPECT. This perfusion defect pattern has been described with varying frequency from 12 to 59% of SPECT patients with documented significant LMS stenosis in retrospective analyses [9, 11, 12]. This perfusion defect pattern was seen in just 8 LMS patients (30%) by CMR and 2 patients (7%) by SPECT in our study. The low diagnostic yield specific for LMS disease may be due, in part, to distal and bifurcation LMS lesions, which may have a differential effect on myocardial perfusion to the LAD and LCx territories, resulting in underestimation of LMS disease. Furthermore, although a visual or QCA reported stenosis of 50% of the LMS is deemed significant by convention, not all 50% coronary stenoses are haemodynamically significant when assessed by invasive fractional flow reserve (FFR) [25]. In addition, a myocardial perfusion abnormality consistent with LMS disease may be less apparent in the presence of coronary collateralisation, or flow-limiting stenosis in the right coronary artery (i.e. 3-vessel disease). However, these haemodynamic factors do not account for the differential detection rates of CMR and SPECT (overall 81% vs. 59% for detection of CAD). The phenomenon of “balanced ischaemia” in multivessel disease potentially leads to an underestimation of disease, in SPECT this effect is reported with variable frequency [26, 27]. In this context, CMR has been shown to have an advantage over SPECT to detect perfusion defects (in multivessel disease) due to a higher spatial resolution [28, 29]. Furthermore, multi-vessel disease has been shown to not be significantly associated with false negatives in CMR [30].

Wide interobserver variability for visual severity of stenoses of the LMS have been reported [31, 32]. In our study QCA was used to determine the severity of angiographic stenoses, as per the CE-MARC study protocol [13, 14]. In this context, there is a potential limitation of the invasive reference standard; however FFR and intra-vascular ultrasound are only recommended as adjuncts in LMS disease assessment in current guidelines and revascularisation decisions are, for the mainstay, based on severity of angiographic stenosis [33, 34].

Additional diagnostic aids have been proposed to improve the sensitivity of SPECT for the diagnosis of LMS disease. TID of the left ventricular cavity in response to stress has been identified to be a strong predictor of cardiac events [35], reflecting global subendocardial ischaemia or stress-induced left ventricular dysfunction from left main or three vessel disease [36]. Increased right ventricular radiotracer uptake has also been independently associated with LMS disease, with a 60% increase from 0.33 ± 0.07 at rest to 0.51 ± 0.07 with stress in LMS patients (*p* < 0.001 compared to controls) [37, 38]. When non-perfusion markers of widespread ischaemia are used alongside perfusion data, the proportion of patients with LMS stenosis identified by SPECT increased from 56 to 83% in one study [9]. In our population, however TID was seen less frequently, with no significant difference in right ventricular uptake between LMS patients and controls suggesting limited discriminatory value. These markers were used for SPECT analysis in this study, but to date have not been used as standard in CMR, and were not prospectively evaluated here.

This study also examined the utility of quantitative CMR perfusion as a potential approach to account for balanced myocardial hypoperfusion that theoretically limits visual analysis in LMS or 3-vessel disease. Other studies have shown that quantitative estimation of myocardial perfusion reserve by CMR over visual analysis improved sensitivity from 74 to 88% and specificity from 58 to 90% for patients suspected to have coronary artery disease, but not confined to LMS [39]. The Fermi deconvolution method used in our study has been shown to perform as well as any other model for the detection of CAD [16]. Patel et al. identified increased ischaemia burden by quantitative perfusion methods using Fermi deconvolution over qualitative assessment as severity of coronary disease increased in patients undergoing perfusion CMR with multi-vessel disease [40]. The value of quantitative CMR analysis for LMS lesions has not been previously detailed. In our study, global MBF was the best quantitative marker and showed high sensitivity and specificity (78 and 85% respectively) for the diagnosis of LMS disease. Quantitative perfusion analysis however was not significantly better than visual CMR perfusion analysis, suggesting that visual perfusion analysis is sufficient to detect heterogeneities in myocardial contrast distribution in LMS disease, a finding supported by dedicated analysis of false-negative CMR [30]. Furthermore, our results suggest there is little additive value to be gained from the quantification of rest perfusion when quantitation of stress perfusion is performed.

### Limitations

Given the low prevalence of LMS disease, the numbers in this prospective study are limited. In our study SPECT analysis did not use attenuation correction; however this was not routine practice when the study was performed [41]. We did not use FFR as our invasive reference standard, however we did use QCA in line with the main CE-MARC paper. The pulse sequence used for perfusion imaging in CE-MARC was not fully optimised for quantitative analysis as it used a single preparation pulse for all three slices and a relatively high contrast agent dose. This may have led to a lower performance of quantitative analysis in this study compared to recent approaches. The lack of a completely linear arterial input function measurement for MBF analysis, with the assumption that concentration is linearly related to signal intensity will result in an overestimate of absolute myocardial blood flow. However, *post-hoc* correction based on baseline signal intensity values would introduce noise into the measurements that could reduce diagnostic accuracy [42]. Furthermore, studies comparing dual-bolus and uncorrected single bolus myocardial blood flow estimates have not shown significant differences in diagnostic accuracy [43]. Our diagnostic accuracy values agree well with other studies in the literature, suggesting that these limitations have not significantly impacted on our findings.

## Conclusion

This study shows that visual stress perfusion CMR had higher diagnostic accuracy than SPECT to detect significant LMS or LMS equivalent disease. Quantitative perfusion CMR by Fermi-constrained deconvolution had similar performance to visual CMR perfusion analysis.

## Abbreviations

AUC: Area under the curve
CAD: Coronary artery disease
CE-MARC: Clinical Evaluation of MAgnetic Resonance imaging in Coronary heart disease
CMR: Cardiovascular magnetic resonance
FFR: Fractional flow reserve
LAD: Left anterior descending coronary artery
LCx: left circumflex coronary artery
LGE: late gadolinium enhancement
LMS: Left main stem
LV: Left ventricle/left ventricular
MBF: Myocardial blood flow
MPR: Myocardial perfusion reserve
QCA: Quantitative coronary angiography
ROC: Receiver operating characteristic
SPECT: Single-photon emission computed tomography
SSS: Summed stress score
TID: Transient ischaemic dilatation
